# Notes and News

**Published:** 1893-09-09

**Authors:** 


### NOTES AND NEWS.

COLLECTED AND COMMUNICATED.

IT appears that the scientific application of electricity for the purpose of executing criminals does not always unfortunately fulfill the humane intentions of its inventors. An American convict was recently subjected to terrible and unavailing tortures for one hour, through the failure of the current supplied to effect its object. We have had certain accidents in the course of our system of hanging, but anything so prolonged as this we feel sure has not occurred.

A MEMBER of the Committee of the Manchester Lifeboat Saturday Fund makes a special appeal to other centres to join in this movement. His words are especially directed to young men who, he states, have been very largely instrumental in the formation of branches in many directions, and who have carried out the work with energy. The movement is popular with all classes, and a very small amount of exertion only is necessary to secure the universal observance of the Lifeboat Saturday.

AT the July meeting of the Church of England Sanitary Association, held at the Church House, Westminster, the Rev. J. R. Byrne, H.M. Inspector of Schools, brought forward for discussion the question of "The Effects of Posture upon the Health of School Children," and showed to demonstration the evils of the existing forms of bench and desk. Curvature of the spine, and serious injury to the eyes have resulted from the positions induced by the uncomfortable seats provided in schools of all kinds. In Mr. Byrnes' opinion, such a condition of things having lasted so long can only be explained by the fact that education has, until recently, been conducted on "the greatest misery principle," with the idea that bodily discomfort conduced to mental development. Reform is sadly needed in this direction, and we are glad to see that an excellent school chair and desk, invented by the Princess of Waldeck, mother to H.R.H. the Duchess of Albany, can be obtained from the National Society for the Education of the Poor, Westminster.

AN amusing plaint comes from a lady bather, with which all who have suffered from the miseries of an ordinary bathing machine will duly sympathise. The writer of the letter in the *Daily Graphic* pathetically urges the inconveniences of the present approved arrangements for ladies bathing at seaside watering places, and suggests the wisdom of adopting the more sensible customs of our Continental neighbours on this point. We cordially agree with "Incognita," and wish her all success in her efforts to reform the seaside habits of the British public.

ONE of the latest pieces of advice by which the public would assist that unfortunate "man and his donkey," the Asylums Board, is that floating fever hospitals should be adopted. Certainly this would get over the vexed question of land sites, and in face of the harassing opposition they endure, one could hardly blame the Board for adopting the suggestion could moorings be found. Since, however, the suggestion has nothing else to recommend it, and everything to condemn it, we trust the authorities will never be driven to this extremity.

A NEW wing has recently been added to the Morley House Convalescent Home, at St. Margaret's Bay, near Dover, and on Saturday last the Mayor and Mayoress of Dover, Sir William and Lady Crundall, and a large number of visitors, inspected the new buildings. These have been erected at a cost of about £4,500, including furnishing, and the number of beds in the home has been increased from 36 to 80. There are special wards for members of the police force, for men engaged in the printing trade, &c., and another ward has been endowed by Mr. Lyle, a sugar merchant, for the use of his workmen. A particular feature in the new wing is the splendid day-room, 60 feet by 30.

A PICTURESQUE entertainment, in the shape of a children's open-air fancy dress ball, has just been given by Sir Thomas and Lady Wright at Leicester in aid of the Children's Hospital in that town. A similar fête last year was most successful, and it is proposed, should the results this year be equally satisfactory, to make it an annual event. The grounds of Abbey Park were charmingly illuminated, and the quaint and pretty dresses of the children made the scene a very attractive one. Sir Thomas and Lady Wright gave a most cordial welcome to their guests, some six thousand in all, and it is to be hoped that the affair will prove to have been as much of a success financially as it undoubtedly was socially.

A NEW wing at the Porthcawl Convalescent Home has just been opened by Miss Talbot, who, as well as her family, has most generously benefited the institution. The Home, which practically serves the whole of South Wales, and Glamorganshire in particular, was provided about 25 years ago for the accommodation of 42 patients, the beds, with the exception of six, being reserved for males. To-day, principally through the generosity and untiring exertion of Miss Talbot, the provision for female patients now equals that for the males. The extension has been an expensive one, and over £1,000 deficiency on the building account appeals to further generosity. The Home is beautifully situated and stands in its own grounds.

